# Teledentistry in the Management of Patients with Dental and Temporomandibular Disorders

**DOI:** 10.1155/2022/7091153

**Published:** 2022-04-09

**Authors:** Giuseppe Minervini, Diana Russo, Alan Scott Herford, Francesca Gorassini, Aida Meto, Cesare D'Amico, Gabriele Cervino, Marco Cicciù, Luca Fiorillo

**Affiliations:** ^1^Multidisciplinary Department of Medical-Surgical and Odontostomatological Specialties, University of Campania “Luigi Vanvitelli”, 80121 Naples, Italy; ^2^Department of Maxillofacial Surgery, Loma Linda University, Loma Linda, CA 92354, USA; ^3^School of Dentistry Department of Biomedical and Dental Sciences and Morphofunctional Imaging, University of Messina, Via Consolare Valeria, 1, 98125 Messina, Italy; ^4^Department of Dentistry, University of Aldent, 1000 Tirana, Albania

## Abstract

Telemedicine is a subunit of telehealth, and it uses telecommunication technology, video, digital images, and electronic medical records to allow the exchange of clinical information and images over remote distances for dental consultation, diagnosis, and treatment planning. Dental clinical practice requires face-to-face interaction with the patients, and therefore, during the COVID-19 pandemic, it has mostly been suspended. In this view, teledentistry offers the opportunity to continue dental practice, avoiding the face-to-face examination that put patients and healthcare professionals at infection risk. Teledentistry encompasses several subunits such as teleconsultation, telediagnosis, telemonitoring, and teletriage. To date, there are several experiences described in literature that suggest that teledentistry could be applied to support traditional care of different oral diseases. However, there are some issues that need to be addressed. Reimbursement concerns, costs, license regulations, limits in physical examinations, and expert equipment are principal issues that should be overcome in telemedicine and in teledentistry. In this narrative review, we provide an overview of the different teledentistry approaches in the care of patients with dental and temporomandibular disorders, as well as discussing the issues that need to be addressed to implement this approach in clinical practice.

## 1. Introduction

Telemedicine being a subunit of telehealth uses telecommunication technology, video, digital images, and electronic medical records to allow the exchange of clinical information and images over remote distances for dental consultation, diagnosis, and treatment planning [1–4].

On March 11, 2020, the World Health Organization (http://www.who.it) recognized COVID-19 as a pandemic. The symptoms of COVID-19 are like those of influenza and include fever, dry cough, hyposmia/anosmia, and shortness of breath [1]. Most of the patients suffer from mild symptoms while a small proportion will develop a severe disease [2].

In today's circumstances of the ongoing COVID-19 pandemic, the main aim is to limit person-to-person contact.

This need has given a boost to the research in telemedicine to continue the management of different diseases remotely, especially for chronic conditions [5–8].

Dental clinical practice requires face-to-face interaction with the patients, and therefore, during the COVID-19 pandemic, it has mostly been suspended [9].

In this view, teledentistry offers the opportunity to continue dental practice avoiding the face-to-face examination that put at risk of infection both patients and health professionals.

Teledentistry encompasses several subunits such as teleconsultation, telediagnosis, telemonitoring, and teletriage [9].

Teledentistry can take one of the three forms: asynchronous (store and forward approach), synchronous (real-time interaction), and mobile health care services (manage and track dental health conditions or promote healthy behaviors using mobile technology).

The temporomandibular disorders (TMD) during this period increased notably due to the emotional stress [10]. The TMDs include several disorders [11] that are categorized as intra-articular (within the joint) or extra-articular (involving the surrounding musculature and other structures) [12, 13]; the etiology is associated with several factors including trauma [14], emotional stress, parafunctional habits, muscle dysfunction, discal or bone morphology alteration [15], inflammatory, and congenital causes [12, 16].

Approximately only 5%-10% of the population will experience a TMD of significance and seek professional assistance at some point in their lives. So many patients live with temporomandibular disorders or manage them autonomously [8, 17]; for this reason, the web and telemedicine can be useful for patient education and self-care management.

In this narrative review, an overview of the different teledentistry approaches has been provided. It could be addressed to the care of patients with oral disease and TMD in particular, with the perspective to implement this approach in clinical practice.

## 2. Methods

The literature search was conducted in the Scopus, Web of Science, and PubMed electronic databases. Document type was limited to articles written in English, without time restrictions.

The search terms included “telemedicine” OR “teledentistry” OR “telemedicine” OR “teleconsultation”, OR “telediagnosis” OR “teletriage” OR “telemonitoring” combined with “temporomandibular disorder”, OR “orofacial pain” OR “bruxism” OR “dental caries”.

The database search was further supplemented with a hand search of relevant articles in the reference lists.

### 2.1. Article Screening

Article screening was conducted independently by GM and DR, both with expertise in clinical management and treatment of temporomandibular disorders. First, duplicate citations were removed. Then, the two authors independently reviewed the retrieved article by title and abstract of each citation to determine its suitability for inclusion. In the initial scan, articles were excluded if they were clearly outside the aim of the review. Next, the authors reviewed the full text of potentially relevant studies (Table 1). The same process was used in conducting the hand search of reference lists. The authors' decisions to include/exclude each article were compared. Discrepancies were discussed, and finally an agreement was reached.

### 2.2. Teleconsultation

Teleconsultation is the most common form of teledentistry; it helps in reducing nonurgent patient referrals [18]. It reduces the number of referrals from primary health centers to higher centers by >45% and reduces burden on busy health care facilities [19].

Remote consultation can be performed via applications for instant messaging (WhatsApp, Telegram, Instagram, SMS, and Messenger) or video calling (Google Meet, Skype, Facetime, and WhatsApp).

Petruzzi and De Benedittis [20] explored the use of WhatsApp in the process of oral diagnosis and revealed that 82% of the remote consulted cases agreed with the clinicopathological diagnosis, suggesting the reliability of this approach.

Ignatius and Perälä [21] performed a 13-month study to evaluate the reliability and feasibility of videoconferencing in making diagnosis and treatment plans for patients requiring prosthetic or oral rehabilitation treatment. 29 subjects took part to the study (24 patients and 25 health professionals). A diagnosis could be made in 24 out of the 27 teleconsultations performed. All health professionals were satisfied for the quality of the process. Further, the study revealed that the technology investments necessary for this approach were relatively modest. The authors concluded that teleconferencing can be easily applied for diagnosis and treatment planning for patients requiring prosthetic or oral rehabilitation treatment.

Salazar-Fernandez and colleagues [22] explored in a multicenter nonrandomized clinical study the effectiveness of a store-and-forward telemedicine system (SFTMS) to manage patients with temporomandibular joint disorders (TMJD). They compared the effectiveness of a store-and-forward telemedicine system with standard care for the selection, diagnosis, and treatment of patients with TMJD referred. The two main findings were that they found similar effectiveness for both systems of consultation and treatment of TMJD and that with SFTMS, a significant reduction of waiting times was achieved. The authors concluded that one of the most relevant advantages of the store-and-forward telemedicine system as a management tool is the reduction of lost working time for patients and the low cost which is especially applicable to patients who live far from the center where they receive the appropriate treatment.

A recent study explored the reliability of a remote protocol for the assessment of patients with TMJD. The primary outcome was the diagnosis of myalgia and arthralgia. The video communication examination was compared to the standard examination in 16 individuals. There was a high to perfect agreement between the standard examination and the remote protocol for the diagnoses of both myalgia and arthralgia [23].

Overall, teleconsultation for the remote assessment of patients with different oral conditions appears to be feasible and reliable with a high degree of accuracy.

### 2.3. Telediagnosis

Telediagnosis makes use of technology to exchange intraoral and radiographic images and data to diagnose an oral lesion [24, 25].

A Brazilian study [26] reported the experience of 89 health providers with the use of EstomatoNet, telediagnosis software. Enrolled subjects submitted comprehensive requests for clinical information and photographs of oral requests via a cloud platform. Specialized oral medicine teleconsultants received the data, conveyed a diagnostic hypothesis, and conveyed management recommendations. Teleconsultations resulted in 42.9% of the patients referred to a specialist, 23.6% of the patients addressed to total biopsy, and 16.2% to follow-up. After the teleconsultation, the need for the patients to face-to-face consultation reduced from 96.9% to 35.1%.

Further, teledentistry offers acceptable reliability for the initial diagnosis of caries in children, as revealed by AlShaya and colleagues [27]. They explored the reliability of mobile teledentistry in the diagnosis of dental caries in children without the use of radiographs. Six pediatrics dentists examined a total of 57 cases using only the images sent to them via a cloud-based platform. Study results revealed a greater sensibility rather than specificity of this approach in dental caries diagnosis; however, both are higher than 80%. Further, the authors found a higher reliability of teledentistry in primary teeth than in permanent teeth.

Another study compared the performance of two smartphone camera and a conventional camera with that of a face-to-face clinical examination for the diagnosis of caries at different stages of progression in deciduous molars. The authors found that the use of a smartphone camera is feasible to detect extensive caries lesions; however, photographic images are not a reliable method for accurately detecting initial and moderate caries lesions [28].

The usefulness of teledentistry was explored also in the assessment of the oral malignant lesions. Sunny and colleagues explored the clinical utility and efficacy of a telecytology system in combination with the artificial neural network-based risk stratification model for early diagnosis of oral potentially malignant and malignant lesion. The telecytology platform, CellScope, was compared to the conventional cytology and showed an overall accuracy of 85% with no difference between the two approaches in detection of oral lesions. The study determines the reliability of telecytology for remote diagnosis of potentially malignant and malignant lesion [29].

### 2.4. Teletriage

Teletriage uses technology to supplement or replace elements of the patient interaction to screen patients remotely and determine the patient's condition and the care needed. It involves the proper, safe, and timely collection of patient symptoms via a smartphone by specialists.

Among US children, the most prevalent infectious diseases are dental caries. National surveys have revealed that minority-group children not only are disproportionately affected by dental caries for poor nutrition but also have limited access to oral health care for high costs and geographical distances [30, 31].

In an ongoing noninferiority, randomized, controlled study, Estai et al. aim to compare routine and teledental pathway of dental care in children aged 4–15 years. All the enrolled patients will receive first a standard examination and then will be photographed using a smartphone. Subjects in the control group will receive the results of the face-to-face examination and advice on the dental care pathway to take whereas subjects in the teledental group will receive the results based on the evaluation of the images. Decay experience and proportion of children becoming caries active will be used as the primary outcome. This project has the objective of prioritizing high-risk children and providing them with a fast treatment pathway, avoiding unnecessary referrals and travels [30].

The University of Rochester's Eastman Institute for Oral Health (EIOH) developed some projects aimed at exploring the feasibility and reliability of asynchronous and synchronous teledentistry to screen and diagnose oral disease. In 2004, EIOH conducted a first pilot project to assess the feasibility of the store and forwarded method (asynchronous teledentistry) to screen dental caries in preschool children [32]. All the subjects screened underwent dental examination. First, they underwent a face-to-face examination by a calibrated dental examiner and then a teledentistry imaging evaluation by an assistant. After two weeks, the images collected during the teledentistry evaluation were evaluated by the first examiner to determine the presence of caries. The diagnostic quality of the teledentistry evaluation was assessed by comparing the results of the face-to face examination (considered the gold standard) to the images obtained using the camera. Study findings reported a sensitivity of 100% and a specificity of 81%. No difference between the asynchronous teledentistry examinations and the face-to face evaluation performed by a calibrated dental examiner was observed, demonstrating the potential for teledentistry to substitute the in-person visual/tactile examination by a dentist or dental hygienist.

In 2010, EIOH started a synchronous teledentistry program in collaboration with the Finger Lakes Community Health (FLCH) to diagnose and manage Medicaid-eligible children with oral disease (mainly dental caries) [31]. FLCH is a federally qualified health center with multiple locations that serves people in the Finger Lakes region of New York State.

When a child seen in one of the FLCH clinics was identified as having the need of a pediatric dentistry, a teledentistry appointment was scheduled. During the teleconsultation, the telepresenter systematically showed the pediatric dentist views of the hard and soft intraoral tissues. After the evaluation, clinical findings and treatment recommendation were discussed with the parents. At the time of the article publication, over 850 rural pediatric patients were seen remotely via a live video teledentistry module [31]. A preliminary review revealed that the rates of treatment completion were higher than those observed in the initial record that was made before starting the remote synchronous evaluation. These findings support the hypothesis that teledentistry consultations could be a practical and potentially cost-effective way to facilitate the use of appropriate treatment pathways and to increase oral health care use when treating complex pediatric dental cases.

Brucoli et al. suggested the use of teleradiology as a useful tool in triaging of maxillofacial trauma patients from the peripheral center to their main trauma center [33]. 467 trauma patients with maxillofacial fractures from the hospitals of a northern Italian region were triaged and managed by the tempore telemedicine system. Surgical indication was suggested for 68 patients, whereas surgery was considered contraindicated for 223 patients. A clinical assessment was considered necessary for 176 patients for the establishment of eventual indications for surgery. Following clinical assessment, the absence and presence of surgical indications were confirmed in all 223 and 68 patients, respectively. These data suggested that teleradiology could be a reliable method to triage trauma patients from peripheral hospitals for the correct referral to a maxillofacial trauma hub center.

### 2.5. Telemonitoring

To monitor the progress of dental patient treatments, frequent in-person examination is needed. Telemonitoring is aimed at replacing the face-to-face visits with virtual visits for regular monitoring of treatment outcomes and disease progression [18]. In a recent pilot study during the pandemic, Giudice and colleagues described the advantages of using teledentistry for the management of patients with oral diagnosis. In this pilot study, patients were divided into two groups: patients with urgent conditions (group U) and patients in follow-up (group F). Patients were instructed to send photos through a messaging service (WhatsApp). Almost all the patients sent photos on the established evening. None of them sent more photos than the number that was established by the protocol. The study revealed that teledentistry appeared to be a promising tool in the remote management of surgical and nonsurgical patients, especially reducing costs and waiting times [34].

## 3. Discussion and Conclusion

The available data suggest that teledentistry can be applied to support traditional care of different oral diseases.

However, there are some issues that need to be addressed. Reimbursement concerns, costs, license regulations, limits in physical examinations, and expert equipment are principal issues that should be overcome in telemedicine and in teledentistry. One of the main challenges is represented by refund; until the reimbursement is not officially and legally recognized, it will slow down development of teledentistry. Reimbursement for medical aid could be available in some cases providing limited compensation for dental professionals in their dental practices. Although there are still some problems concerning the refund, the use of telemedicine services has reduced the cost of healthcare treatment for patients. So many studies explore cost-effectiveness of teledentistry.

The cost and benefits of visual examination conducted by a dentist in rural areas with a teledentistry approach were compared by Mariño and colleagues who concluded that teleconsultation was a lower cost service model compared to the in-person model of care [35].

A cost-minimization analysis was conducted to compare the costs of teledentistry with outreach visits and hospital visits; the authors reported that teledentistry can help in reducing oral health inequalities making cost savings significantly higher in remote regions, even with additional costs [36].

Salazar-Fernandez and colleagues conducted another cost-minimization study for the management of patients with TMJD suggesting that the use of telemedicine can shorten the delay in treatment initiation, avoiding or reducing the loss of productivity [22].

Teledentistry increases access to dental services for those who live in rural areas and allows quick and easy connections between doctors and patients. Indeed, teledentistry could be particularly useful for people living in underserved areas as it allows continuous monitoring without the need to reach specialists in urban areas. However, in underserved areas, the lack of high-speed broadband access may account for the inability to reach underserved populations [37]. Many software applications have been proposed during these months, some involve the use of complex devices for communication, others instead only the use of a smartphone, others in a combined way the use of a smartphone plus a tool that allows you to correctly capture the images to be communicated to the clinician. One of the latter software is represented by DentalMonitoring®. Dental monitoring is a new scan box that now is going to be used in a considerable number of private clinics. This allows remote monitoring of the patient, through the use of a smartphone and device for the correct upload of the photos and the standardization of the latter. The software possessing an algorithm can already offer useful information to the clinician regarding the clinical case. This is an important tool for both orthodontic and TMD patients but also for remote monitoring of other problems [38–47].

Future studies are needed to identify the best intervention protocols, efficacy, safety, feasibility, and benefit-cost ratio to promote the use of reimbursed teledentistry approaches in clinical settings.

## Figures and Tables

**Table 1:** Summary of the studies discussed.

Authors	Title	Subunit	Modality	Oral disease	Primary outcome reached	Strengths	Limitations
Petruzzi and De Benedittis	WhatsApp: a telemedicine platform for facilitating remote oral medicine consultation and improving clinical examinations	Teleconsultation/telediagnosis	Mobile health care services	Malignant and benign oral lesions	Yes	Comparison with the clinicopathologic diagnosis/sample size	No regulation and standardization of quality images
Ignatius and Perälä	Use of videoconferencing for consultation in dental prosthetics and oral rehabilitation	Teleconsultation	Synchronous	Prosthetic or oral rehabilitation treatment	Yes	—	Small sample size
Salazar-Fernandez et al.	Telemedicine as an effective tool for the management of temporomandibular joint disorders	Teleconsultation	Asynchronous	TMJD	Yes	Multicenter/sample size	—
Exposto et al.	Remote physical examination for temporomandibular disorders	Teleconsultation/telediagnosis	Synchronous	Myalgia and arthralgia due to TMJD	Yes	Comparison with standard examination	Small sample size
Carrard et al.	Telediagnosis of oral lesions in primary care: the EstomatoNet Program	Telediagnosis	Asynchronous	Malignant and benign oral lesions	—	Sample size	No comparison with standard examination
AlShaya et al.	Reliability of mobile phone teledentistry in dental diagnosis and treatment planning in mixed dentition	Telediagnosis	Asynchronous	Dental caries	Yes	Examination of the oral cavity based on WHO criteria/comparison with standard examination	Restricted age group 6–9 years (no data on permanent teeth)
Kohara et al.	Is it feasible to use smartphone images to perform telediagnosis of different stages of occlusal caries lesions?	Telediagnosis	Asynchronous	Dental caries	Yes	Comparison with standard examination	All the photographic images were taken by the same operator
Sunny et al.	A smart tele-cytology point-of-care platform for oral cancer screening	Telediagnosis	Asynchronous	Oral malignant lesions	Yes	Comparison with conventional cytology	Small sample size
Estai et al.	Teledentistry as a novel pathway to improve dental health in school children: a research protocol for a randomised controlled trial	Teletriage	Asynchronous	Dental care	Study ongoing	Comparison with standard examination	—
Kopycka-Kedzierawski et al.	Dental screening of preschool children using teledentistry: a feasibility study	Teletriage	Asynchronous	Dental care	Yes	Comparison with standard examination	Restricted to preschool children
Kopycka-Kedzierawski and McLaren	Advancement of teledentistry at the University of Rochester's Eastman Institute for Oral Health	Teletriage	Synchronous	Dental care	Ongoing	Sample size	No comparison with standard examination
Brucoli et al.	The use of teleradiology for triaging of maxillofacial trauma	Teletriage	Asynchronous	Maxillofacial trauma	Yes	Comparison with standard examination	—
Giudice et al.	Can teledentistry improve the monitoring of patients during the Covid-19 dissemination?	Telemonitoring	Mobile health care services	Oral diseases	N/A	—	No comparison with standard examination

TMJD: temporomandibular joint disorder; N/A: not applicable; WHO: World Health Organization.

## Data Availability

Data is available on request to the corresponding author.
